# The Impact of Rationalization and Upgrading of Industrial Structure on Carbon Emissions in the Beijing-Tianjin-Hebei Urban Agglomeration

**DOI:** 10.3390/ijerph19137997

**Published:** 2022-06-29

**Authors:** Runde Gu, Chunfa Li, Dongdong Li, Yangyang Yang, Shan Gu

**Affiliations:** 1 School of Management, Tianjin University of Technology, Tianjin 300384, China; rundegu@stud.tjut.edu.cn (R.G.); 183103402@stud.tjut.edu.cn (D.L.); 2 Tians Engineering Technology Group Co., Ltd., Shijiazhuang 050035, China; gushan@tianjushi.com

**Keywords:** the Beijing-Tianjin-Hebei urban agglomeration, rationalization of industrial structure, industrial structure upgrading, carbon emission

## Abstract

Carbon dioxide mainly comes from industrial economic activities. Industrial structure optimization is an effective way to reduce carbon dioxide emissions. This paper uses the panel data of 13 cities in the Beijing-Tianjin-Hebei urban agglomeration from 2006 to 2019, uses the Theil index to calculate the industrial structure rationalization index, and uses the proportion of industrial added value to calculate the industrial structure upgrade index. By constructing the STIRPAT model, this paper quantitatively analyzes the impact of industrial structure rationalization and upgrade on carbon emissions. The results show that the rationalization and upgrading of industrial structure in the Beijing-Tianjin-Hebei urban agglomeration significantly inhibit carbon emissions. Compared with the rationalization of the industrial structure, the upgrading of industrial structure in the Beijing-Tianjin-Hebei urban agglomeration has a better effect on carbon emission reduction. For the Beijing-Tianjin-Hebei urban agglomeration, government expenditure on science and technology can promote the upgrading of industrial structure to a certain extent, thereby reducing carbon emissions. There is a big gap between the industrial structure development level of Hebei province and that of Beijing and Tianjin. Finally, based on the conclusion, this paper puts forward the policy enlightenment of promoting the optimization process of industrial structure and reducing carbon emissions of the Beijing-Tianjin-Hebei urban agglomeration.

## 1. Introduction

Since the 21st century, industrialization and urbanization have significantly increased energy consumption. They contribute to the rapid growth of carbon emissions, exacerbating global climate change and threatening the natural environment and human health [1]. Environmental protection and sustainable development of regions and industries have become hot issues of concern worldwide. Controlling carbon emissions and developing a low-carbon economy have become common choices [2]. China’s carbon emissions account for about 1/3 of the world’s total carbon emissions, and it is the world’s largest carbon emitter. Pressure from public opinion and environmental problems at domestic and foreign levels make the Chinese government’s demand for carbon emission reduction increasingly urgent [3]. Of China’s total carbon emissions, urban carbon emissions and industrial carbon emissions account for a large proportion. Improving the regional industrial economy while reducing carbon emissions is crucial to sustainable economic development [4].

The Beijing-Tianjin-Hebei region is one of China’s three major urban agglomerations and plays a pivotal role in the national economic territory. The total GDP of this region in 2020 was CNY 8.6 trillion, accounting for about 8.5% of the country. At the same time, Beijing-Tianjin-Hebei is China’s main heavy industrial base. In 2019, the total energy consumption of Beijing-Tianjin-Hebei was 481 million tons of standard coal, accounting for 9.84% of the national energy consumption, of which Hebei Province ranked third with 325 million tons of traditional coal. A large amount of energy consumption and exhaust emissions have caused severe air pollution, making Beijing-Tianjin-Hebei one of the areas with the sharpest contradiction between economic development and resources and the environment in China [5]. To this end, in the Outline of the Beijing-Tianjin-Hebei Coordinated Development Plan, breakthroughs should be made in critical areas such as industrial upgrading and transfer, ecological and environmental protection in the Beijing-Tianjin-Hebei region, and the deployment is planned. Under the deep integration of Beijing-Tianjin-Hebei coordinated development, the two-way coordination of economic growth and environmental protection makes it inevitable to strengthen regional ecological governance and optimize industrial structure. Therefore, optimizing the regional industrial structure is very important to reducing the carbon emissions of the Beijing-Tianjin-Hebei urban agglomeration and even the whole country.

The rationalization and upgrading of industrial structure determine the regional division of labor status, industrial ecological layout and low-carbon sustainable development prospects of the Beijing-Tianjin-Hebei. It is an essential guarantee for the high-quality and green development of the Beijing-Tianjin-Hebei region and is highly valued by the national and local governments. It is worth noting that there are hierarchical differences in the industrial structure of Beijing-Tianjin-Hebei, which are the key areas of China’s industrial structure adjustment. We aim to reveal the relationship between regional industrial structure and carbon emissions and put forward countermeasures and suggestions that can improve the quality of regional economic development and provide a demonstration effect for other regions to achieve the low-carbon result. Therefore, this paper takes 13 cities in the Beijing-Tianjin-Hebei urban agglomeration as the research object and selects relevant panel data from 2006 to 2019. Then, we apply the STIRPAT model to quantitatively analyze the impact of rationalization and upgrading of industrial structure in the Beijing-Tianjin-Hebei urban agglomeration on carbon dioxide emissions. In general, the main contributions of this paper are as follows:

(1) This study takes the Beijing-Tianjin-Hebei urban agglomeration as an overall region to explore the relationship between its industrial structure and carbon emissions, which positively deepens the joint industrial development and spatial layout in the Beijing-Tianjin-Hebei urban agglomeration.
(2) This study subdivides the industrial structure variables into two independent variables—industrial structure rationalization and industrial structure upgrade—to better understand the impact of industrial structure optimization on carbon emissions. Other variables include population, per capita GDP and government expenditure on science and technology, and the variables are considered comprehensively. In addition, the robustness test of carbon emission intensity as a replacement variable of total carbon emissions confirms the credibility of the results. It expands the impact on carbon emission intensity.
(3) This study aims to provide a reference for the formulation of carbon emission reduction policies from the rationalization of industrial structure and upgrading of industrial structure. It is of great significance for China to deepen the optimization of industrial structure in the Beijing-Tianjin-Hebei region and the top-level design of urban low-carbon sustainable development for local governments to grasp the correct policy direction and academic circles to keep up with the needs of regional industrial green development.

The remainder of the article is organized as follows: Section 2 gives the literature review and combs and evaluates the existing research. Section 3 presents a detailed discussion of our model’s construction and methodology. Section 4 introduces the research area, data sources and processing. Section 5 is empirical analysis, mainly including unit root test, cointegration test, empirical results analysis, reality analysis and robust test. The final section gives the results and discusses some policy implications.

## 2. Literature Review

**Figure 1:**
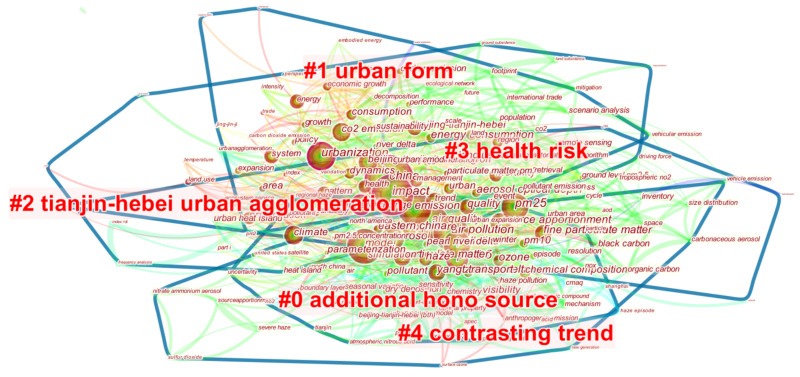
Keyword clustering map.

In this section, in the literature exploration of “Beijing-Tianjin-Hebei (Jing-Jin-Ji)” research published in SCI journals, we used the CiteSpace software to conduct keyword clustering analysis on 884 pieces of literature in the Web of Science database [6]. We selected the LLR algorithm to generate a keyword clustering map (Figure 1).

As shown in Figure 1, China, CO_2_ emission, air pollution, air quality, urbanization, Beijing-Tianjin-Hebei region, PM_2.5_ and so on have a large dot area in the map and occupy a core position. The clustered keywords are classified into “additional HONO sources” “urban form” “Tianjin-Hebei urban agglomeration” “health risk” and “contrasting trends”, as shown in Table 1.

According to the bibliometric data and clustering results, the research emphasis of the Beijing-Tianjin-Hebei region is the subject of urban agglomeration air pollution control. Existing studies mainly focus on the generation [7,8], impact [9,10] and treatment [11,12] of air pollution in the Beijing-Tianjin-Hebei urban agglomeration.

The global over-exploitation of natural resources and the increasing fossil energy consumption make the air pollution crisis increasingly severe. The concept of green and sustainable development has been integrated into all levels of economy and society, and a low-carbon economy has become an inevitable choice. The focus is on reducing high-carbon energy consumption and CO_2_ emissions. As the essential industrial bases, Beijing, Tianjin and Hebei have carried out extensive and in-depth studies on energy consumption and carbon emissions in this region using the factor decomposition method, index decomposition method and input-output method [13,14,15]. Xue et al., (2021) used the EBM model of unexpected output to measure the city-level carbon emission efficiency of the Beijing-Tianjin-Hebei region from 2007 to 2016 [16]; Chang et al., (2020) estimated the carbon emissions of Bohai Rim from 2005 to 2017, and evaluated the impact of energy consumption structure on carbon emission performance in the region [17]; Bai et al., (2021) concluded that driving force shows that due to the economic transformation of the measurement, emission intensity and production structure dramatically reduces the amount of the Beijing-Tianjin-Hebei region [18]; Wang et al., (2021) developed a multi-scenario factorial analysis and multi-regional input-output model, and multiple scenarios based on direct CO_2_ reduction and final demand mitigation on various industries are examined [19].

**Figure 2:**
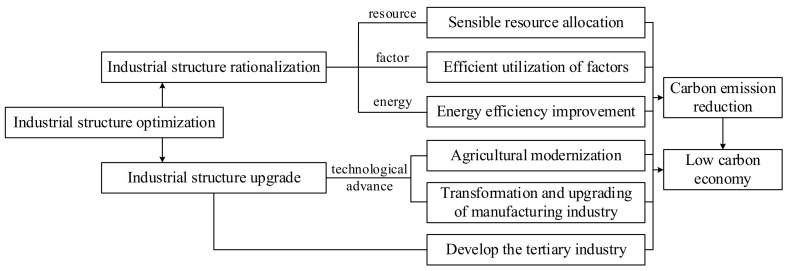
Influence path of industrial structure optimization on carbon emission reduction.

With the acceleration of industrial transformation and upgrading, scholars began to pay attention to the relationship between industrial structure and carbon emissions. They proved that industrial structure adjustment significantly impacts carbon emissions [20,21,22,23]. The impact path is shown in Figure 2. However, Wu et al., (2018) found that the adjustment of the industrial structure affects changes in response to shifts in different regions [24]. The Beijing-Tianjin-Hebei region is in a critical industrial structure transformation and upgrading period. Scholars have carried out in-depth studies on the impact of urban industrial structure on carbon emissions by adopting various methods. Mi et al., (2015) constructed an input-output model to evaluate and prove that Beijing’s industrial structure adjustment has excellent potential for energy conservation and carbon reduction [21]; Zhu et al., (2020) established a multi-objective optimization model to analyze the impact of industrial restructuring in Beijing on carbon emissions and energy consumption during 2018–2020 [25]; Siqin et al., (2022) constructed an expanded Cobb–Douglas production function and proved the one-way causal relationship between industrial structure and CO_2_ emissions in North China through econometric analysis. Tianjin, Hebei and Shanxi showed reverse causality, while Beijing and Inner Mongolia did not have causality [5]. Meanwhile, this study verifies the results of Wu (2018).

Scholars have conducted many discussions on the factors affecting carbon emissions, and relevant studies have been relatively systematic. The impact of the industrial structure on carbon emissions research is also rising increasingly. However, there are still some aspects that can be extended, including: (1) from the angle of geography, existing research for urban geographical units and urban agglomeration as the leading economic regionalization in China, there is little research on overall regional urban agglomeration, its industrial structure and the relationship between carbon emissions; (2) many factors affect CO_2_ emissions, such as industrial structure, per capita GDP and scientific and technological level, etc. However, most existing studies only involve individual factors. Few articles study the impact of rationalizing and upgrading industrial structure on carbon emissions as independent variables. Therefore, this paper takes the Beijing-Tianjin-Hebei urban agglomeration as the research object and selects the panel data from 2006 to 2019 to quantitatively analyze the impact of rationalization of industrial structure, industrial structure upgrading, population number, per capita GDP and government science and technology expenditure on carbon dioxide emissions.

## 3. Model Construction and Calculation Method

### 3.1. Model Construction

In the initial model construction, Holdren (1974) proposed the IPAT environmental impact equation in 1971 [26]. The basic form is as follows.

(1) I=P×A×T

In Formula (1), *I* is the environmental impact, *P* is the population, *A* is the economic prosperity, and *T* is the technical level. This formula reflects the relationship between economy and environment, but it cannot reflect the non-proportional impact of various influencing factors on the environment. On this basis, Dietz and Rosa (1997) proposed the STIRPAT model in 1997 [27]. The model is as follows.

(2) $$I=aP^{b}A^{c}T^{d}e$$

In Model (2), *a* is the constant term, *b*, *c*, *d* is the elasticity of environmental impact, and *e* is the error term. The representation of *I*, *P*, *A* and *T* are the same as the Model (1). To eliminate heterogeneity, all variables are taken as logarithms. As shown in Model (3).

(3) lnI=lna+blnP+clnA+dlnT+lne

The regression coefficient in model (3) reflects the elastic relationship between the explanatory and explained variables. The STIRPAT model has good expansibility, allowing it to join the other control variables for analysis and calculation. To research the impact of industrial structure on carbon emissions in the Beijing-Tianjin-Hebei urban agglomeration, considering the degree of rationalization of industrial structure and the degree of upgrading industrial structure. The Model (3) is expanded to Model (4).

(4) $$\ln C_{it}=\alpha_{it}+\beta_{1}\ln POP_{it}+\beta_{2}\ln GDP_{it}+\beta_{3}\ln TE_{it}+\beta_{4}\ln H_{it}+\beta_{5}\ln G_{it}+\varepsilon_{it}$$

In Model (4), *i* and *t* are the sample size and time, respectively; *C* is the total urban carbon emissions; *POP* is the total population of the city, expressed by the city’s year-end registered population; *GDP* is the city’s economic prosperity, defined in terms of per capita Gross Domestic Product (GDP); *TE* is the technical level, expressed by financial expenditure on science and technology; *H* is the rationalization degree of industrial structure, which is measured by the rationalization index of industrial structure; *G* is the degree of industrial structure supererogation, which is measured by the industrial structure supererogation index; *α* is the constant term; *ε* is the random disturbance term.

### 3.2. Calculation Method

The urban carbon emission measurement mainly includes liquefied petroleum gas, natural gas, coal gas, electricity consumption and transportation carbon emission. Due to the lack of measurement data on transportation, carbon emission accounts for a small proportion of the total carbon emission. Based on the methods of Ren et al. (2020) [28], this study calculates energy consumption through natural gas, liquefied petroleum gas, gas, electricity consumption and heat energy. As for the total heat, coal-based heat demand is the main reason for carbon dioxide emissions. Therefore, the full heat is first converted into raw coal and then multiplied by the carbon dioxide emission coefficient of raw coal to measure carbon dioxide emissions. The calculation method of each city’s total carbon dioxide emission is shown in Formula (5) [29].

(5) $$C_{i}^{t}=\sum_{j}C_{ij}^{t}=kE_{1}+vE_{2}+rE_{3}+uE_{4}+\theta E_{5}$$

In Formula (5), *C_i_^t^* is the total energy-related CO_2_ emissions in sub-sector *i* in year *t*; *C_ij_^t^* is the CO_2_ emissions based on fuel type *j* in sub-sector *i* in year *t*; *E*_1_, *E*_2_, *E*_3_, *E*_4_ and *E*_5_ are natural gas, liquefied petroleum gas, coal gas, electricity consumption and heat energy (coal consumption), respectively; *k*, *v*, *r*, *u* and *θ* are energy correlation coefficients, from IPCC Guidelines for National Greenhouse Gas Emission Inventory 2006.

The rationalization of industrial structure refers to the aggregation quality between industries, reflects the coordination degree and sufficient utilization degree of resources between industries, and measures the coupling degree of factor input structure and output structure. This paper refers to the research results of Gan (2011) and Zhang (2018), who used the Theil index to calculate the industry rationalization index [30,31]. Theil index represents regional economic disparity, and the value is proportional to the degree of distinction. The formula is as follows:

(6) $$H=\sum_{i=1}^{n}\left(\frac{Y_{i}}{Y}\right)\ln\left(\frac{Y_{i}}{L_{i}}/\frac{Y}{L}\right)$$

In Formula (6), *i* is industry; *n* is the number of industry sectors; *Y* is the gross urban product; *L* is the number of employees. *Y_i_/Y* is the output structure; *Y_i_/L_i_* is industrial productivity; *Y/L* is the total industrial production. When the Theil index is 0, the economy is at an equilibrium and the industrial structure is optimal. Theil index is inversely proportional to the degree of industry rationalization. The smaller the value, the more reasonable the industrial structure.

The industrial structure upgrading is the process of establishing and realizing a high-efficiency industrial structure. It refers to the process in which the focus of economic development or industrial structure shifts from the primary industry to the secondary and tertiary industries, which marks the level and stage of economic growth [32]. This paper adopts the research conclusion of Gan (2011). It takes the ratio of the added value of the tertiary industry to the added value of the secondary industry as an indicator to measure the advanced degree of industrial structure [30].

## 4. Overview of the Study Region and Data Sources

### 4.1. Study Region

**Figure 3:**
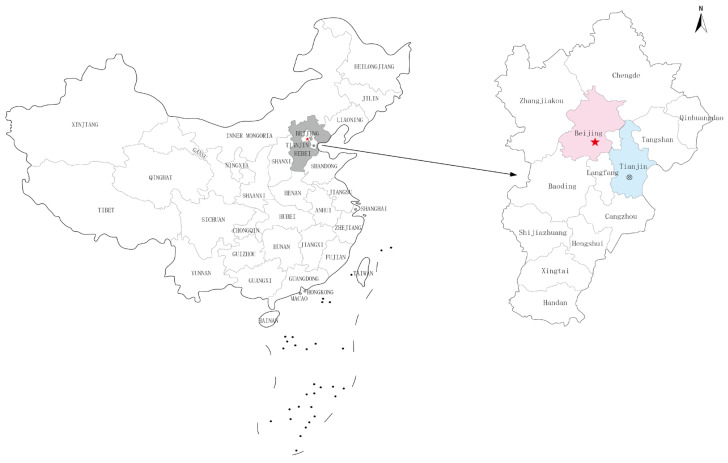
Location map of Beijing-Tianjin-Hebei Region.

The Beijing-Tianjin-Hebei urban agglomeration is a world-class urban agglomeration led by Beijing and Tianjin, including Shijiazhuang, Baoding, Tangshan and other 13 cities (Figure 3). It is located in the heart of Bohai Rim in Northeast China, with a total area of 218,000 km^2^ and a population of 110.3 million. It is the largest and most dynamic region in northern China. It plays an essential pivotal role in the national economy and transportation maps [18]. At the same time, Beijing-Tianjin-Hebei is also a heavy industry base of metallurgy, chemical industry, building materials, automobile manufacturing, machinery manufacturing and other industries with high energy consumption and high pollution [15]. Since 2013, the Beijing-Tianjin-Hebei region has suffered from an extensive and prolonged haze, with PM_2.5_ levels exceeding the standard for a long time, making it a central disaster area for air pollution. The Outline of the Beijing-Tianjin-Hebei Coordinated Development Plan issued in 2015 made industrial upgrading and relocation and ecological and environmental protection the focus of coordinated regional development. Since then, the industrial structure of Tianjin has been adjusted, from the initial “two, three, one” to “three, two, one” step distribution. Among the three regions, Tianjin is in the alternate stage of industrial structure and has entered the later stage of industrialization. Beijing has entered the post-industrialization period, and the tertiary industry shows a high development trend due to the removal of non-capital functions. Hebei province is still in the middle of industrialization. The secondary industry still occupies a significant market share and is the leading industry in its economic development. The tertiary industry is in an upward growth trend, but there is still a big gap between Beijing and Tianjin. The upgrading of the industrial structure of the Beijing-Tianjin-Hebei urban agglomeration reflects the re-matching of resource elements. The development of the tertiary industry will further promote the transformation and upgrading of the industrial structure and reduce energy consumption and environmental pollution while improving the regional economy.

### 4.2. Data Sources and Processing

**Figure 4:**
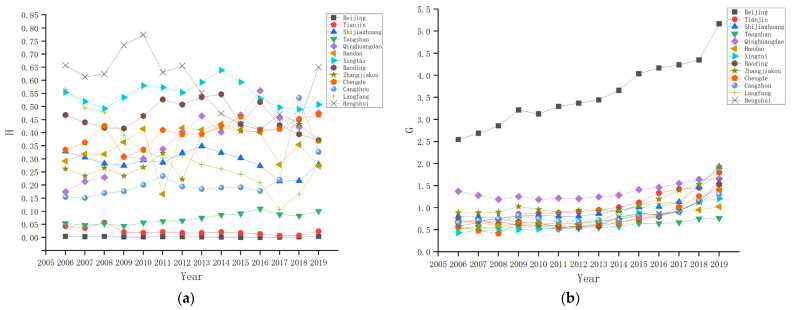
(**a**) The rationalization index of industrial structure in each city; (**b**) the upgrading index of industrial structure in each city.

This paper conducts a quantitative analysis on the impact of the rationalization and upgrading of the industrial structure of the Beijing-Tianjin-Hebei urban agglomeration on carbon dioxide emissions, taking 13 cities such as Beijing, Tianjin, and Shijiazhuang as the research objects and selecting data from 2006 to 2019. In this paper, the total urban population, per capita GDP, financial expenditure on science and technology, the whole urban GDP, the number of employees, and industrial output value are all from the China City Statistical Yearbook, China Energy Statistical Yearbook, municipal statistical yearbook and statistical bulletin [33]. To eliminate heteroscedasticity, the main variables are treated with a logarithm. The description and statistics of relevant variables are shown in Table 2. The industrial structure rationalization index in each city is shown in Figure 4a, and the industrial structure upgrade index in each city is shown in Figure 4b.

## 5. Empirical Analysis

### 5.1. Unit Root Test

In the process of panel data modeling, to ensure the stability of variable data for economic analysis and avoid “pseudo regression”, this paper performs a unit root test on variable data before regression analysis. A unit root test is commonly used to test the stationarity of time series variables, mainly including LLC, IPS, Fisher-ADF and Fisher-PP methods. LLC method assumes that the unit root process of each section individual is the same, and other techniques allow the unit root process of each section individually to be different. This paper uses Stata17.0 software (StataCorp LLC, College Station, TX, USA) to carry out the above tests on variables, and the results are shown in Table 3.

When the probability value is less than 0.05, the test sequence rejects the original hypothesis, indicating that the variable does not have a unit root. It can be seen from Table 3 that all the actual variables fail the unit root test, and all pass the four-unit root tests after the first-order difference of the original variables. The result indicates that the first-order difference sequence of the actual variables is stable and the test conditions are full.

### 5.2. Co-Integration Test

The co-integration test aims to test whether there is a stable equilibrium relationship between variable sequences. This article is based on the panel data of the Engle and Granger co-integration test method by using the technique of Kao test analysis to analyze whether there is a co-integration relationship between the explanatory variable and explained variable. We set the null hypothesis that there is no co-integration relationship among all variables. If the test results reject the null hypothesis, it is proved that there is a co-integration relationship between variables. We use Stata17.0 software to conduct the co-integration test on the above variables. The results are shown in Table 4.

According to the co-integration test results in Table 4, the corresponding *p* values of all t values of the Kao test are less than 0.01. The data show that at the confidence level of 1%, the statistical results of the Kao test of the original variable are very significant, which rejects the null hypothesis that there is no co-integration relationship. The above results show a co-integration relationship between the actual variables. Therefore, the regression analysis can be performed on the econometric Model (4).

### 5.3. Regression Results

According to Model (4), the impacts of rationalization and upgrading of industrial structure on carbon emissions of the Beijing-Tianjin-Hebei urban agglomeration were measured. In this paper, the F test was used to judge whether a fixed-effect model or random effect model should be used; the Breusch–Pagan test was used to evaluate whether a random effect model or mixed effect model should be used; the Hausman test was used to judge whether fixed-effect model or random effect model should be used. The results show that all models are suitable for the fixed effect model.

The rationalization index of industrial structure and the upgrading index of industrial structure are added to the model, respectively, and then they are added into the model together. To test the robustness of the model, the variable *TE* was added to the model as a control variable for analysis. The regression results are shown in Table 5.

According to the regression results, the R^2^ values are about 0.7, indicating that the fitting degree of the regression model is good, and the overall significance of the parameter estimation results is high. Therefore, the constructed econometric model can better explain the relationship between the rationalization of industrial structure, the upgrading of industrial structure, and carbon emission. The result accords with the expectation of this paper.

### 5.4. Empirical Results Analysis

In Table 4 regression results, the Models (1) and (2) only join the industrial structure rationalization index, the Models (3) and (4) only join the industrial structure upgrade index, Models (5) and (6) enter the industrial structure rationalization index and the industrial structure upgrade index as explanatory variables simultaneously. The six models were divided into three groups, including Models (1) and (2), Models (3) and (4), and Models (5) and (6). In each group, two models were distinguished as to whether to add control variables. This paper analyzes the regression results and data. It can be concluded that:

(1) The regression results of Models (1), (2), (5) and (6) show that the rationalization of industrial structure of the Beijing-Tianjin-Hebei urban agglomeration is positively correlated with carbon emissions. According to the regression data, every 1% decrease in industrial structure rationalization index will decrease carbon emissions by 0.057% to 0.068%. It can be known from the previous analysis that the smaller the industrial structure rationalization index is, the more reasonable the industrial structure is. Therefore, the rationalization of industrial structure in Beijing-Tianjin-Hebei urban agglomeration significantly inhibits carbon emissions. The reason is that the unreasonable industrial structure creates the uneven distribution of resources, resulting in the waste of resources and an increase in energy consumption. Ultimately, carbon emissions increase.
(2) The regression results of Models (3), (4), (5) and (6) show that the upgrading of industrial structure of Beijing-Tianjin-Hebei urban agglomeration is negatively correlated with carbon emissions. According to the regression data, every 1% increase in industrial structure upgrade index will decrease carbon emissions by 0.171% to 0.194% on average. The growth of the upgrading industrial index indicates that the proportion of the added value of the tertiary industry increases, and the carbon emission of the tertiary industry increases. However, at the same time, the proportion of the added value of the secondary industry decreases, and the carbon emission decreases. As the average carbon emission intensity of the secondary industry is higher than that of the tertiary industry, the total carbon emission falls. Therefore, improving the advanced level of industrial structure of Beijing-Tianjin-Hebei urban agglomeration can inhibit carbon emissions.
(3) Two variables, the industrial structure rationalization index and the industrial structure upgrade index, are added to Models (5) and (6), then the regression results are still significant. It shows that the overall regression result is stable. There is only a tiny change in the industrial structure rationalization index when the control variable TE is added. The same is true in Models (1) and (2). In Model (4), the addition of TE reduces carbon emissions by 0.09% more than in Model (3) when the industrial structure upgrading index increases by 1%. Similarly, Model (6) reduces carbon emissions by 0.06% more than Model (5). It reflects that the government’s increased financial expenditure on science and technology will help promote the upgrading of industrial structure and reduce carbon emissions. To sum up, the upgrading of industrial structure and rationalization of industrial structure in the Beijing-Tianjin-Hebei urban agglomeration all help to reduce regional carbon emissions.
(4) According to the model regression results, the per capita GDP of the Beijing-Tianjin-Hebei urban agglomeration has a significant positive effect on carbon emissions. The regression data shows that for every 1% increase in per capita GDP, the average carbon emissions increase by 0.420–0.471%. The regression results accord with the general economic law. However, the impact of population change on carbon emissions is not significant. It may be due to the slight change in the number of urban populations in Beijing, Tianjin, and Hebei and many employed people in the primary industry in Hebei Province.
(5) From the overall regression results, the impact of every 1% change in industrial structure rationalization index on carbon emissions is 0.057% to 0.068%, and the impact of every 1% change in industrial structure upgrade index on carbon emissions is 0.171% to 0.194%. It shows that the effect of industrial structure upgrading on carbon emission reduction is better. Additionally, it proves that the removal of carbon emissions of Beijing-Tianjin-Hebei urban agglomeration depends more on upgrading industrial structure. In the future, the government should pay attention to regional industrial transformation and industrial upgrading, promote the development of producer services and strategic emerging industries, and improve the advanced level of industrial structure.

### 5.5. Reality Analysis

**Figure 5:**
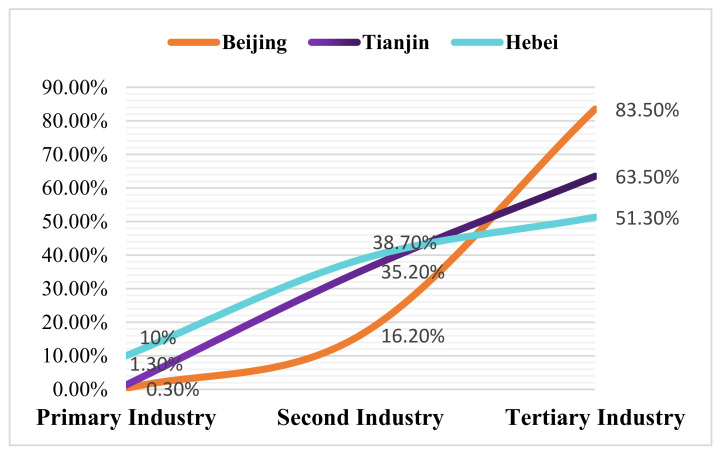
Proportion of industries in Beijing, Tianjin and Hebei (2019).

In the development of the Beijing-Tianjin-Hebei region, Beijing has played a leading role, while Tianjin has shown excellent development potential. The industrial structure of the two cities shows a gradient distribution of “three, two, and one”. As shown in Figure 5, the tertiary industry accounted for 83.5% and 63.5%, respectively, in 2019. In the past, Hebei province served Beijing in the economic development process of The Beijing-Tianjin-Hebei urban agglomeration. Although Hebei province has the largest area, the most abundant resources, and the highest overall GDP scale, it has the lowest per capita production efficiency and the lowest per capita GDP, which has become an economic disconnection belt. Compared with Beijing and Tianjin, Hebei province has different levels of industrial structure development. Among the three major industries, the primary industry still accounts for a large proportion of about 10%. The secondary industry is mainly steel and machinery manufacturing, which has caused severe environmental pollution while increasing the regional economy and has once become a central disaster area for pollution prevention and control. The tertiary industry is on a gradual growth trend, accounting for 51.3% in 2019, but there is a big gap between the other two regions. To rationalize its development, it is still necessary to adjust the industrial structure and gradually upgrading from primary and secondary to tertiary industries. With the rapid growth of the modern service industry, the Beijing-Tianjin-Hebei urban agglomeration will further promote the coordinated development of industries, reduce energy and environmental constraints, and reduce the total carbon emissions. However, it should be recognized that only when the tertiary industry development level of the three places remains relatively the same can a better regional economic development ecology be created.

### 5.6. Robust Test

Carbon emission intensity is the proportion of urban carbon emission level and regional economic development level. It is one of the essential indicators to measure carbon emission and reflect the impact of urban economic development on environmental pollution. To verify the econometric analysis results of the relationship between the industrial structure and carbon emissions in the Beijing-Tianjin-Hebei urban agglomeration, whether the signs and significance of regression coefficients change with parameter setting. Based on the above considerations, this paper uses the carbon emission intensity index as a substitute index for the total urban carbon emissions to test the robustness of the model. The method of measuring carbon emission intensity in this paper is as follows.

(7) $$CD=\frac{C}{GDP_{\mathrm{real}}}$$

In Formula (7), *CD* is the carbon emission intensity of a city, *GDP* is the real gross regional product, and *C* is the total carbon dioxide emission of a city, which obtained data are calculated using Formula (5). The regression results are shown in Table 6.

As shown in Table 6, the coefficient signs and significance of regression results do not change significantly. It indicates that the relationship between the rationalization of industrial structure, the upgrading of industrial structure and carbon emissions of the Beijing-Tianjin-Hebei urban agglomeration still exists, which confirms that the regression result obtained from the overall data is robust.

## 6. Conclusions

This paper calculates the industrial structure rationalization index, industrial structure upgrading index and carbon emission of 13 cities in the Beijing-Tianjin-Hebei urban agglomeration from 2006 to 2019. Then, it uses the STIRPAT model to analyze the impact of industrial structure rationalization and upgrading of the Beijing-Tianjin-Hebei urban agglomeration on carbon dioxide emission. According to the regression results of the research model, we draw the following conclusions:

(1) The rationalization of industrial structure in the Beijing-Tianjin-Hebei urban agglomeration significantly inhibits carbon emissions. Specifically, every 1% decrease in the industrial structure rationalization index will decrease carbon emissions by 0.057% to 0.068% on average.
(2) Improving the advanced level of the industrial structure of the Beijing-Tianjin-Hebei urban agglomeration can inhibit carbon emissions. Specifically, every 1% increase in the industrial structure upgrading index will decrease car-bon emissions by 0.171% to 0.194% on average.
(3) For the Beijing-Tianjin-Hebei urban agglomeration, government expenditure on science and technology can promote the development of industrial structure upgrade to a certain extent, thereby reducing carbon emissions. Specifically, after increasing financial expenditure on science and technology, every 1% increase in the industrial structure upgrading index will decrease carbon emissions by 0.06% to 0.09% on average.
(4) Compared with the rationalization of the industrial structure, the upgrading of industrial structure in the Beijing-Tianjin-Hebei urban agglomeration has a better effect on carbon emission reduction.
(5) Compared with Beijing and Tianjin, there are differences in the development level of industrial structure in Hebei Province. It is necessary to adjust the industrial structure and promote the development level of tertiary industry in the three places to maintain a relatively same level.

Based on the above research conclusions, in order to improve the rationalization and upgrading of Beijing-Tianjin-Hebei Industrial structure, reduce carbon emissions and ensure the stable development of a low-carbon economy in the Beijing-Tianjin-Hebei urban agglomeration, this paper puts forward the following suggestions.

(1) The rationalization of industrial structure can realize the rational allocation of production factors. It achieves the effect of reducing energy consumption and carbon emissions by reducing the waste of resources. Therefore, the government of Beijing, Tianjin and Hebei should promote the transformation and upgrading of enterprises by providing preferential policies and financial support. The government should guide enterprises to increase investment in research and development of environmental protection technologies and energy-saving industries, further optimize the allocation of internal resources in enterprises, realize the improvement of the level of rationalization within the industry, and promote carbon emission reduction from the source of production. In addition, cities should also pay attention to the complementary advantages and coordinated development of industries, improve the utilization rate of regional resources, and establish regional carbon emission reduction cooperation to reduce the overall carbon emissions in the region.
(2) The upgrading of industrial structure aims to reduce carbon emissions by applying digital and low-carbon technologies in traditional industries and increasing the share of the tertiary industry. Hebei Province should use digital technology to drive the transformation and upgrading of traditional fields such as agriculture and manufacturing, guide and encourage enterprises to develop and utilize low-carbon technologies, further eliminate outdated production capacity, and guide the transfer of excess resources to the service industry, thereby reducing urban carbon emissions. For cities with a high share of the tertiary industry, such as Beijing and Tianjin, upgrading their advanced industrial level mainly relies on high-tech and service industries with high added value and low carbon emissions. Therefore, Beijing and Tianjin should focus on transforming traditional service industries, reducing carbon emissions such as transportation, and developing emerging service industries driven by technological innovation. At the same time, the Beijing-Tianjin-Hebei urban agglomeration should expand emerging industries, such as new energy, new materials, energy conservation and environmental protection, and steadily build an energy-saving and consumption-reducing industrial system to create natural conditions for reducing carbon emissions.
(3) Government spending on science and technology can improve regional green innovation capabilities, promote regional industrial structure optimization, and achieve low-carbon industrial transformation and development. Therefore, the governments of Beijing, Tianjin and Hebei should increase their spending on science and technology, attract knowledge, talents, technology and other innovative elements to enterprises, promote the development of high-tech industries and emerging industries of energy conservation and emission reduction, and improve the advanced level of industrial structure. Enterprises should increase R&D efforts with the support of government spending on science and technology to provide a technical foundation for the development of producer services and consumer services. In addition, the government should set up special funds to introduce significant scientific and technological achievements that can promote the transformation and upgrading of traditional industries and the development of high-tech industries. By implementing the transformation of scientific and technological achievements, the government has consolidated the foundation for optimizing the industrial structure. Increasing government spending on science and technology affects carbon emission reduction by optimizing the industrial structure.
(4) Hebei province should implement a differentiation policy when accepting industries transferred from Beijing and reject enterprises with high energy consumption and heavy pollution. High pollution taxes should be levied on enterprises with moderate energy consumption and pollution to encourage them to reduce pollution emissions. Tax incentives and financial subsidies should be given to enterprises with low energy consumption and low pollution to help them develop rapidly. At the same time, for existing industries, we should speed up the elimination of backward production facilities, reduce the use of fossil energy, and curb the blind development of projects with high energy consumption and high emissions.

However, this paper has some limitations and further research directions to consider. First, this paper builds a static model focusing on the causal relationship between the optimization of the industrial structure of Beijing, Tianjin and Hebei on carbon emissions. However, due to space limitations, it does not explore the mediating or pre-driving factors in this impact pathway. In addition, from the perspective of spatial measurement, it is necessary to analyze the dynamic evolution process and spatial spillover effects of the industrial structure optimization of Beijing-Tianjin-Hebei. The above expansion research is the future research direction of this paper. It can provide detailed and quantifiable research inspiration for Beijing-Tianjin-Hebei to achieve industrial structure optimization and carbon emission reduction goals.

## Figures and Tables

**Table 1:** Keyword cluster comparison.

No.	Cluster Name	Clustering Subcluster (Part)
0	Additional HONO source	Pollution; boundary layer; aerosol radiative effect; atmospheric NO_x_
1	Urban form	Tianjin-Hebei region; regions; taking China; estimating interregional payments
2	Tianjin-Hebei urban agglomeration	Eastern China Tianjin-Hebe region; CO_2_ emission; evaluating urban sustainability
3	Health risk	Surface PM_2.5_; using aerosol; water-PM_2.5_; linkage analysis; fenwei pain
4	Contrasting trend	Winter haze; summertime surface ozone; severe haze events; atmospheric circulations

**Table 2:** Variable description and descriptive statistics.

Variable	Meaning	Average Value	Standard Deviation	Maximum Value	Minimum Value
*C*	Carbon dioxide emissions (10^6^ t)	63.46	31.92	154.00	13.35
*POP*	Total urban population (million)	7.486	3.184	13.97	2.81
*GDP*	Per capita GDP (CNY/person)	43,966	29,612	164,220	11,146
*TE*	Financial expenditure on science and technology (million)	2644	7144	43,342	21.58
*H*	Industrial structure rationalization index	0.305	0.192	0.774459	0.000146
*G*	Industrial structure upgrade index	1.090	0.812	5.169	0.413

**Table 3:** Unit root test results of variable.

Unit Root Test	Variable	LLC	IPS	Fisher-ADF	Fisher-PP
Original	*lnPOP*	−3.0347 ***	−0.1277	36.0216 *	39.4504 **
	*lnGDP*	−5.3142 ***	−1.4475 *	37.5218 *	45.4989 **
	*lnC*	−4.2074 ***	0.1736	16.7423	37.2991 *
	*lnH*	−2.1145 **	−0.2685	33.9075	18.2475
	*lnG*	0.2149	2.4602	24.3220	16.9871
	*lnTE*	−5.9964 ***	−3.4877 ***	80.0884 ***	65.2765 ***
First difference	*lnPOP*	−9.7035 ***	−7.0845 ***	109.4168 ***	128.0388 ***
	*lnGDP*	−11.0732 ***	−6.2828 ***	53.3246 ***	134.0895 ***
	*lnC*	−9.8797 ***	−5.8720 ***	49.1081 ***	108.1036 ***
	*lnH*	−9.2334 ***	−5.3773 ***	63.4136 ***	88.4551 ***
	*lnG*	−6.9195 ***	−4.0900 ***	141.4963 ***	67.8694 ***
	*lnTE*	−13.6008 ***	−7.4961 ***	107.7971 ***	198.2123 ***

Note: *** *p* < 0.01, ** *p* < 0.05, * *p* < 0.1.

**Table 4:** Co-integration test results of variables.

	Statistics	*p*-Value
Modified Dickey–Fuller t	−2.5868	0.0048
Dickey–Fuller t	−2.6560	0.0040
Augmented Dickey–Fuller t	−2.9696	0.0015
Unadjusted modified Dickey–Fuller t	−3.6494	0.0001
Unadjusted Dickey–Fuller t	−3.0925	0.0010

**Table 5:** Regression results analysis.

Variables	Model					
	(1)	(2)	(3)	(4)	(5)	(6)
*lnH*	0.067 *** (3.38)	0.068 *** (3.54)			0.058 ** (2.43)	0.057 ** (2.46)
*lnG*			−0.185 *** (−4.43)	−0.194 *** (−5.65)	−0.171 *** (−3.81)	−0.177 *** (−4.93)
*lnPOP*	−0.203 (−0.64)	−0.163 (−0.45)	0.202 (0.38)	0.175 (0.31)	0.340 (0.81)	0.320 (0.71)
*lnGDP*	0.420 *** (10.25)	0.441 *** (−2.11)	0.471 *** (8.18)	0.441 *** (4.63)	0.458 *** (8.65)	0.438 *** (4.89)
*lnTE*		−0.015 (−0.43)		0.022 (0.71)		0.014 (0.49)
Constant	5.911 *** (9.48)	5.994 *** (9.90)	5.172 *** (5.01)	4.989 *** (5.02)	4.969 *** (6.15)	4.854 *** (6.35)
Observations	182	182	182	182	182	182
R-squared	0.699	0.699	0.713	0.715	0.732	0.732
R^2^-a	0.693	0.692	0.709	0.708	0.726	0.725
F	75.50	146.6	26.20	147.4	87.72	207.7

Note: *** *p* < 0.01, ** *p* < 0.05

**Table 6:** Regression results analysis (robust test).

Variables	Model					
	(1)	(2)	(3)	(4)	(5)	(6)
*lnH*	0.112 *** (5.60)	0.114 *** (6.18)			0.102 *** (4.19)	0.103 *** (4.46)
*lnG*			−0.218 *** (−5.95)	−0.218 *** (−5.81)	−0.194 *** (−5.16)	−0.187 *** (−4.90)
*lnPOP*	−1.026 * (−1.89)	−0.901 (−1.59)	−0.652 (−0.83)	−0.650 (−0.81)	−0.411 (−0.67)	−0.389 (−0.62)
*lnGDP*	−0.604 *** (−17.36)	−0.536 *** (−8.81)	−0.537 *** (−11.79)	−0.535 *** (−9.29)	−0.561 *** (−15.91)	−0.540 *** (−10.10)
*lnTE*		−0.047 * (−1.92)		−0.002 (−0.09)		−0.016 (−0.71)
Constant	−8.269 *** (−7.67)	−8.004 *** (−7.92)	−8.983 *** (−5.83)	−8.967 *** (−6.04)	−9.338 *** (−7.78)	−9.211 *** (−8.15)
Observations	182	182	182	182	182	182
R-squared	0.863	0.865	0.858	0.858	0.879	0.879
R^2^-a	0.861	0.862	0.856	0.855	0.876	0.876
F	130.2	151.8	89.65	82.94	111.9	111.5

Note: Robust t-statistics in parentheses. *** *p* < 0.01, * *p* < 0.1.

## Data Availability

Not applicable.
